# Simultaneous enhancement of magnetic and mechanical properties in Ni-Mn-Sn alloy by Fe doping

**DOI:** 10.1038/srep43387

**Published:** 2017-02-23

**Authors:** Changlong Tan, Zhipeng Tai, Kun Zhang, Xiaohua Tian, Wei Cai

**Affiliations:** 1 College of Applied Science, Harbin University of Science and Technology, Harbin 150080, China; 2 School of Materials Science and Engineering, Harbin Institute of Technology, Harbin 150001, China

## Abstract

Both magnetic-field-induced reverse martensitic transformation (MFIRMT) and mechanical properties are crucial for application of Ni-Mn-Sn magnetic shape memory alloys. Here, we demonstrate that substitution of Fe for Ni can simultaneously enhance the MFIRMT and mechanical properties of Ni-Mn-Sn, which are advantageous for its applications. The austenite in Ni_44_Fe_6_Mn_39_Sn_11_ shows the typical ferromagnetic magnetization with the highest saturation magnetization of 69 emu/g at 223 K. The result shows that an appropriate amount of Fe substitution can really enhance the ferromagnetism of Ni_50_Mn_39_Sn_11_ alloy in austenite, which directly leads to the enhancement of MFIRMT. Meanwhile, the mechanical property significantly improves with Fe doping. When there is 4 at.% Fe added, the compressive and maximum strain reach the maximum value (approximately 725.4 MPa and 9.3%). Furthermore, using first-principles calculations, we clarify the origin of Fe doping on martensitic transformation and magnetic properties.

Ferromagnetic shape memory alloys (FSMAs) have attracted significant attention since a giant magnetic-field-induced strain was first reported in Ni_2_MnGa alloys^1^,^2^,^3^,^4^,^5^,^6^,^7^,^8^,^9^,^10^,^11^,^12^,^13^,^14^,^15^,^16^. The mechanism of Ni-Mn-Ga FSMA is the martensite variant reorientation as a result of magnetic field-induced twin boundary motion^2^,^17^,^18^. Compared with Ni-Mn-Ga FSMAs, the new Mn-rich Ni-Mn-X (X = In, Sn, Sb) FSMAs show a different mechanism of martensitic transformation. This new type alloys exhibit multifunctional properties and the richness and diversity of the physical phenomena due to the magnetic-field-induced phase transformation^5^. Ni-Mn-Sn based Heusler alloys are good candidates for smart magnetic materials since they are cheap, readily available, non-toxic and exhibit first order magnto-structural transition. The magnetic driving force for such metamagnetic phase transformation is provided by the Zeeman energy difference between the two phases, i.e., *E*_zeeman_ = Δ*M* × *H*, where Δ*M* is the saturation magnetization difference between the austenite and martensite and B corresponds to the strength of the applied field^19^. Form the application point of view obtaining a large Δ*M* and the excellent mechanical properties for FSMAs are extremely important. Actually, both mechanical properties and Δ*M* in FSMAs are very sensitive to their chemical compositions^7^,^20^,^21^,^22^. The partial substitution is believed as an effective method to tune the properties for these alloys. For example, the substitution of Cu for Mn in Ni_43_Mn_46−x_Cu_x_Sn_11_ can shift the martensitic transformation to higher temperature^23^, while the substitution of Cu for Ni in (Ni, Cu)_50_Mn_36_Sn_14_ can shift the martensitic transformation to a lower temperature^24^. Feng *et al*. found that the mechanical properties of Ni-Mn-Sn alloys can be dramatically improved by adding Fe^25^,^26^,^27^,^28^,^29^,^30^. However, rare research has been carried out to improve the Δ*M* and mechanical properties at the same time.

Studies have shown that by substituting a small amount of Co for Ni, a local ferromagnetic structure was achieved in the antiferromagnetic matrix and the magnetization of the high temperature phase was effectively enhanced. And a large Δ*M* was obtained^31^. We know that Co and Fe belong to the same group, and they are both magnetic atoms. Moreover, element Fe has larger magnetic moment compared with element Co. It is logically to expect that substituting a small amount of Fe for Ni, may improve the Δ*M* significantly. As to the mechanical property, a similar problem is also found in the Ni-Mn-Ga alloys. To improve ductility, a second ductile γ phase has been introduced by the addition of Co or Fe into Ni-Mn-Ga alloys^32^. At the same time, we expect that substitution of Fe for Ni in Ni-Mn-Sn alloys introduces the second phase and enhances the ductility of the alloys. So base on this idea, we put forward that Ni_50−*x*_Fe_*x*_Mn_39_Sn_11_ would be a kind of magnetic shape memory alloys with favorable mechanical and better magnetic properties. This present paper focuses on the influence of Fe addition in Ni_50−*x*_Fe_*x*_Mn_39_Sn_11_ (*x* = 0, 3, 4, 6 at.%) alloys on the microstructure, martensitic transformation, and magnetic properties. In addition, we use first-principle calculations to investigate the structural, electronic and magnetic properties on substitution of Fe for Ni atom. Comparing the results of these calculations of the martensitic transformation and magnetic properties of the alloy with experimental findings, we clarify the mechanism of the change of martensitic transformation temperature and the improved magnetic properties. Our results suggest that Fe doped Ni-Mn-Sn alloy possesses promising potential application in smart magnetic materials.

## Results and Discussion

### Microstructure

**Figure 1:**
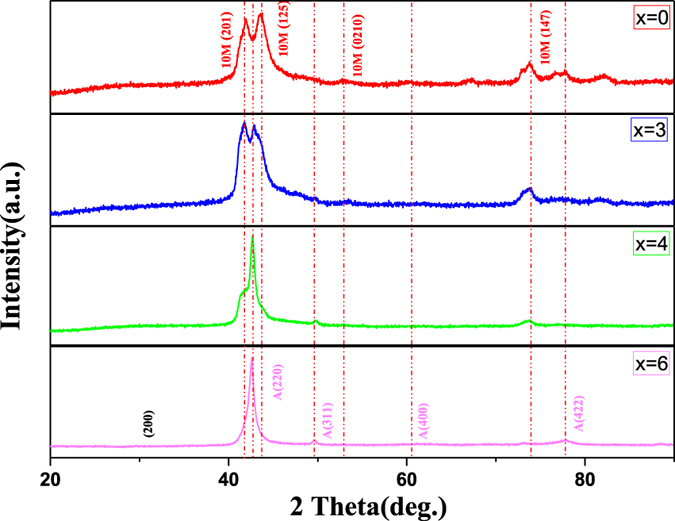
XRD of Ni_50−*x*_Fe_*x*_Mn_39_Sn_11_ alloys at room temperature (**a**) *x* = 0; (**b**) *x* = 3; (**c**) *x* = 4; (**d**) *x* = 6.

Figure 1 shows the X-ray diffraction patterns of Ni_50−*x*_Fe_*x*_Mn_39_Sn_11_ alloys (x = 0, 3, 4, 6 at.%) taken at room temperature. Compared with the reflection of Ni-Mn-Sn alloys reported by Krenke *et al*., it is found that the Ni_50−*x*_Fe_*x*_Mn_39_Sn_11_ alloys (x = 0, 3 at.%) have a 10 M modulated martensitic structure. However, for Fe4 alloy, the typical diffraction peaks from austenite begin to be clearly detected. It indicates that it has coexistence of 10 M martensite and austenite. It should be noted that, with the adding of Fe element, the crystal structure of the alloys has an evident change at room temperature. It transformed into the austenitic structure with high symmetry from martensitic structure with low symmetry. While the Fe6 alloy has a single phase with the crystal structure of cubic *L*2_1_-type at room temperature.

**Figure 2:**
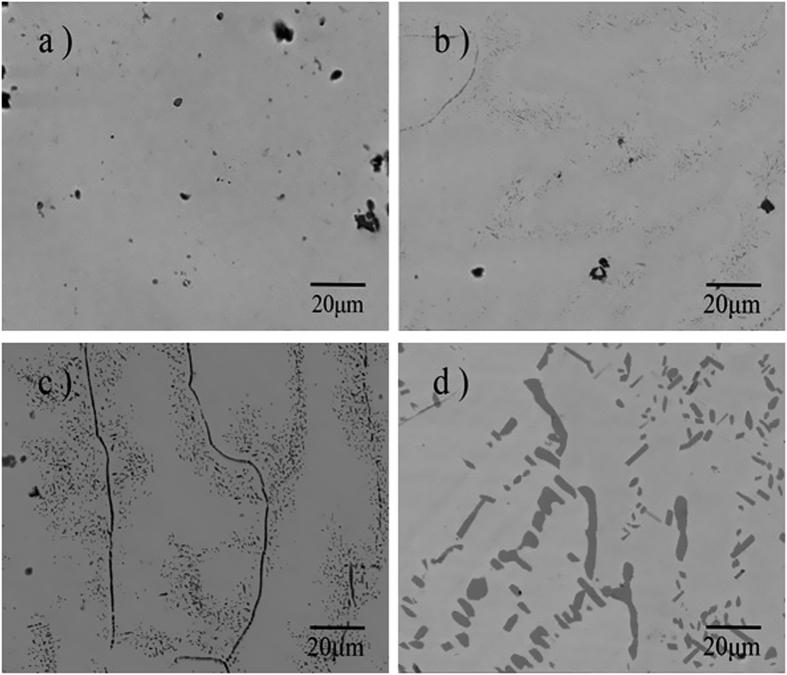
Backscattered electron images of Ni_50−*x*_Fe_*x*_Mn_39_Sn_11_ alloys (**a**) *x* = 0; (**b**) *x* = 3; (**c**) *x* = 4; (**d**) *x* = 6.

Figure 2 shows back-scattered SEM micrographs of the microstructure of the Ni_50−*x*_Fe_*x*_Mn_39_Sn_11_ alloys (*x* = 0, 3, 4, 6 at.%) after homogenization treatment. The Fe0 sample showed a uniform single phase structure without any sign of second phase. A few black spots presented in alloy Fe0 are solidification shrinkage pores formed during ingot casting. These pores are also presented in the other alloys samples. The structure of the Fe3 is essentially identical to that of Fe0. Except for the actual chemical composition of the matrix, Fe4 sample showed a continuous matrix in light contrast and dispersed γ phase particles in dark contrast. The volume proportion of γ phase obviously increased with more Fe content from Fe3 to Fe4. In particular, though the volume proportion of γ phase increased a lot, the morphology of Fe6 was completely different from that of other alloys. This special structure may lead to the poor mechanical properties. The detail of mechanical properties will be discussed in the next section. The composition of the matrix and the second phase is listed in Table 1. From the table, it is seen that the matrix phase of the Fe-doped alloys contained about 39 at.% Mn. The content of Ni decreased continuously from 49.8 at.% to 45.4 at.% with the increasing Fe addition from 0 at.% to 3.1 at.% in the matrix. The γ-phase contains a small amount of Sn but a large amount of Fe.

### Martensitic transformation

**Figure 3:**
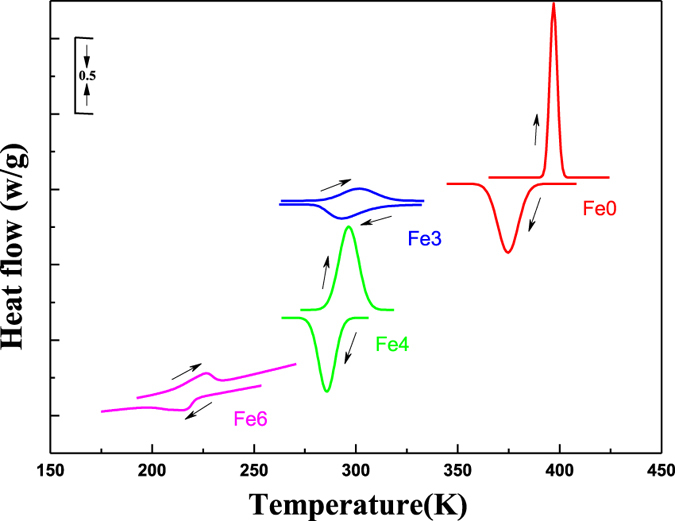
DSC curves of the Ni_50−*x*_Fe_*x*_Mn_39_Sn_11_ alloys.

To investigate the matensitic transformation of Ni_50_Mn_39_Sn_11_ alloys, in which Ni was partly replaced by Fe. Figure 3 presents DSC curves of the Ni_50−*x*_Fe_*x*_Mn_39_Sn_11_ (*x* = 0, 3, 4, 6 at.%) alloys. Next to the features associated with the martensite start *M*_s_, martensite finish *M*_f_, austenite start *A*_s_, and austenite finish *A*_f_ temperatures are also observed. The structure transition temperatures determined from the calorimetry data are indicated with a DSC subscript and are collected in Table 2. The martensitic transformation is clearly observed for the Ni_50−*x*_Fe_*x*_Mn_39_Sn_11_ (*x* = 0, 3, 4, 6 at.%) alloys. We can find that the transformation temperatures decreased with increasing Fe addition in these alloys. The martensitic transformation temperatures are 375, 294, 286, 214 K for Fe0, Fe3, Fe4, Fe6 alloys, respectively. It is seen that the martensitic transformation behavior evolved progressively with the substitution of Fe for Ni in these alloys. On the causes of the martensitic transformation temperature decreases, we make the further theoretical explanation in the last part.

### Magnetic properties

**Figure 4:**
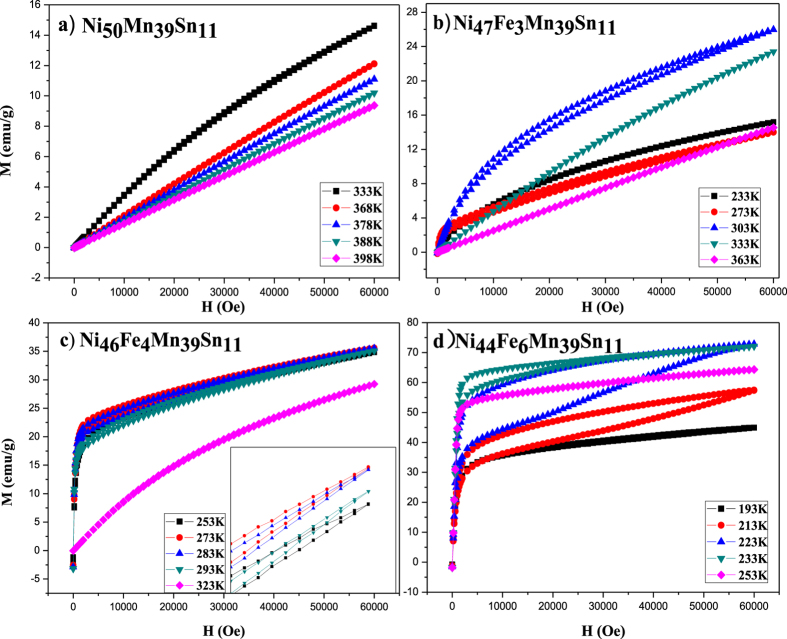
Magnetization curves of Ni_50−*x*_Fe_*x*_Mn_39_Sn_11_ alloys at different temperatures (**a**) *x* = 0; (**b**) *x* = 3; (**c**) *x* = 4; (**d**) *x* = 6.

In order to investigate the magnetic behavior of the alloys at different temperature, we measured the magnetization isotherms in the vicinity of the martensitic transformation temperature region under the magnetic field up to 6 T as shown in Fig. 4.

Figure 4(a) shows the magnetization curves of Fe0 alloy at different temperatures which are all below the martensitic transformation finished temperature (*M*_f_). We can see that the alloy samples in the high temperature region of the martensite phase exhibits paramagnetic or antiferromagnetic behavior. When the temperature decreases, the magnetization curves gradually show the typical ferromagnetic characteristics. This phenomenon is related to the secondary magnetic phase transformation of martensite in Ni_50_Mn_39_Sn_11_ alloy.

Figure 4(b) displays the isothermal magnetization curves of Fe3 alloy tested at temperatures of 233, 273, 303, 333 and 363 K. At a temperature of 303 K, which is 15 K below *A*_f_, the magnetization occurs a rapid increase within the low magnetic field, corresponding to the magnetization of the austenite. With further rising of the magnetic field, the magnetization can hardly be saturated. Figure 4(c) shows the isothermal magnetization curves of Fe4 alloy. It is found that it has the same magnetization behavior with the Fe3 alloy shown in Fig. 4(b).

Figure 4(d) shows that the magnetization of Fe6 was drastically increased and then saturated with magnetic field. At 193 K, which is 21 K below the *A*_s_ temperature, the ferromagnetic martensite showed soft magnetization behavior to nearly saturation at 45 emu/g (at 6 T). And the reverse demagnetization path overlapped with the forward magnetization path. At a temperature of 233 K, which is just above *A*_f_ temperature, the austenite showed a similar behavior of magnetization and demagnetization, but with a higher saturation magnetisation of 69 emu/g. Moreover, from Fig. 4(b), we can see that Fe6 alloy has obvious magnetic hysteresis losses at 223 K. At 223 K, which is 9 K below the *A*_f_ temperature, the sample magnetized to ~48 emu/g at below 1.5 T, corresponding to the magnetization of the martensite. Upon increasing the external field to above 1.5 T, the transformation from the martensite to austenite was induced. The magnetization also nearly reached the saturation magnetization level of the austenite at 6 T, indicating the completion of the MFIRMT. That is, 100% of martensite phase turn to the 100% of austenite phase. When the magnetic field was removed, little austenite phase transformed back to the martensite because the austenite is thermodynamically stable at the testing temperature (223 K).

The Fe6 alloy showed a much higher magnetization in the entire temperature region than other alloys with little Fe addition. This fully shows that an appropriate amount of Fe substitution can really enhance the ferromagnetism of Ni_50_Mn_39_Sn_11_ alloy in austenite. Furthermore, we use first-principles calculations to gain insight into the origin of Fe doping on magnetic properties in Ni-Mn-Sn in the last part.

### Mechanical properties

**Figure 5:**
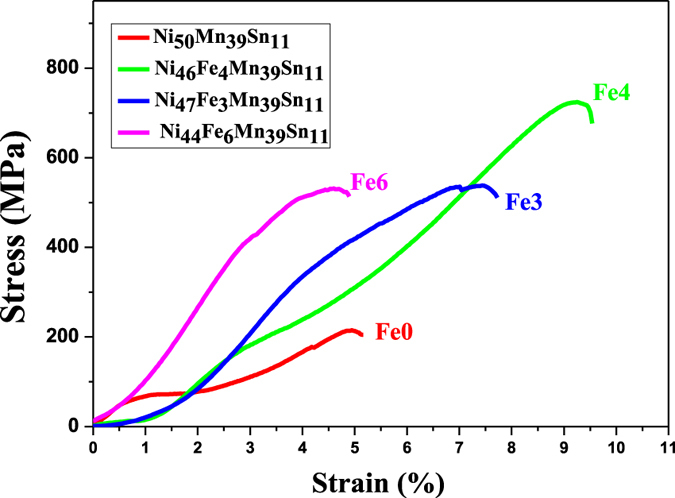
The compressive stress-strain curves of Ni_50−*x*_Fe_*x*_Mn_39_Sn_11_ alloys at room temperature.

In order to obtain the strength and ductility behavior of Ni_50−*x*_Fe_*x*_Mn_39_Sn_11_ (*x* = 0, 3, 4, 6 at.%) alloys. The alloys were loaded at room temperature until fracture in compression as shown in Fig. 5.

As there is no Fe element added, the fracture stress and strain values of Fe0 alloy were only about 215.0 MPa and 4.9% separately. Evidently, the compressive strength and strain of Fe0 alloys were enhanced by addition of Fe. With the increase of Fe content, the mechanical properties were improved gradually. When there is 4 at.% Fe added, the compressive and maximum strain reach the maximum value (approximately 725.4 MPa and 9.3%), which were significantly improved than that of the alloy without Fe addition. with the further increase in Fe content, the compressive strain is decreased largely. It should be noted that, according to the DSC results, Fe6 alloy is austenite while others are martensite initially. Although the second phase formation increased the strength slightly. It also made the alloy more brittle. This clearly indicates that the proper amount of Fe addition significantly improves the compressive strength and the ductility of Ni_50_Mn_39_Sn_11_ alloy.

**Figure 6:**
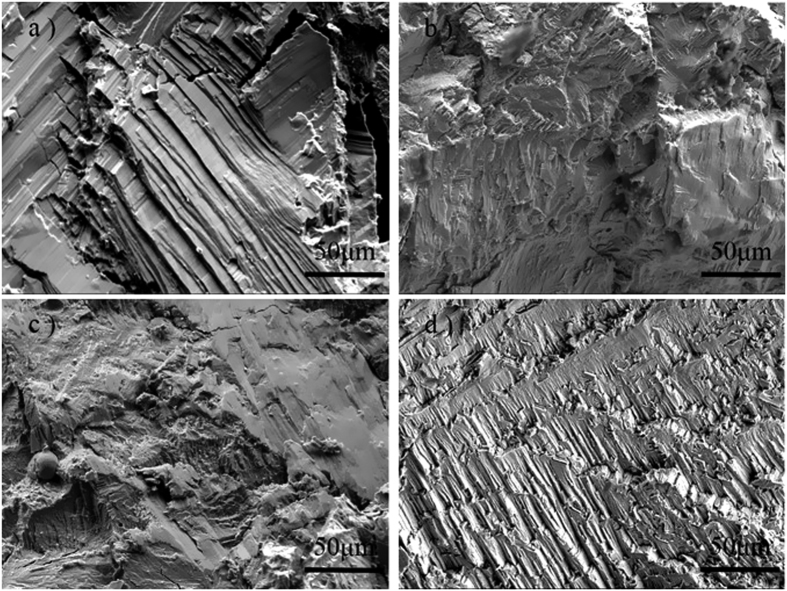
SEM fracture cross-section of Ni_50−*x*_Fe_*x*_Mn_39_Sn_11_ alloys (**a**) *x* = 0; (**b**) *x* = 3; (**c**) *x* = 4; (**d**) *x* = 6.

In order to clarify the fracture mechanism, the fracture morphologies of Ni_50−*x*_Fe_*x*_Mn_39_Sn_11_ alloys after compressing is observed as shown in Fig. 6. Before being doped Fe element, Ni_50_Mn_39_Sn_11_ alloy is brittle, and the fracture of it is typical intergranular crack of intermetallics. As shown in Fig. 6(a), it presents fracture flake and its crystal interface is smooth. For the Fe3 alloy, though the main fracture type is intergranular fracture, some tearing edges are observed, as shown in Fig. 6(b). Thus, the ductility of the alloy is improved. When the Fe content is 4 at.%, a large number of tearing edges appear. The fracture surfaces of this alloy exhibit characteristics of ductile transgranular fracture and plastic deformation occurs before fracturing. As mentioned before, the second phase appears with the Fe addition. We can find some holes on the fracture, which is just because of the loss of the second phase particles during compressive test. All of these make it become the alloy with the best mechanical properties, which is consistent with the results of the compressive stress-strain curves shown in Fig. 6(c). It can be seen from Fig. 6(d) that the fracture of Fe6 has a significantly different with others. It is just because that Fe6 has the structure of austenite phase at the room temperature.

### Origin of Fe doping on magnetic properties and martensitic transformation

**Figure 7:**
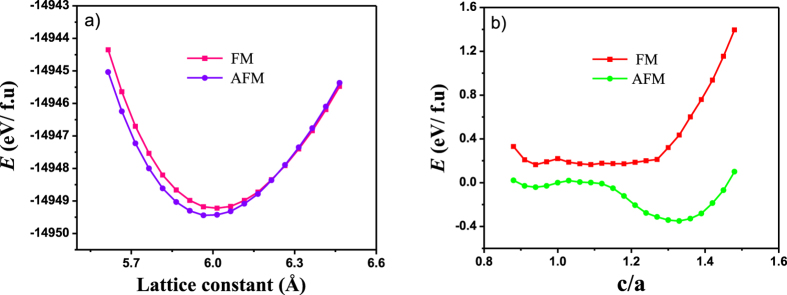
(**a**) Equation of states of Ni_2_Mn_1.5_Sn_0.5_ cubic phases for both parallel and antiparallel magnetic interactions. (**b**) The total energies *E* of Ni_2_Mn_1.5_Sn_0.5_ with a variation of the tetragonal ratio *c*/*a*.

Furthermore, we use first-principles calculations to gain insight into the origin of Fe doping on magnetic properties and martensitic transformation in Ni-Mn-Sn. We considered un-doped Ni_2_Mn_1.5_Sn_0.5_ and 6.25 at.% Fe doped Ni_1.75_Fe_0.25_Mn_1.5_Sn_0.5_, which is close to the content of samples in our experiment, to explore the effect of Fe substitution for Ni. The crystal structure of the austenite phase in Ni-Mn-Sn is *L*2_1_. We first calculate the equation of states of Ni_2_Mn_1.5_Sn_0.5_ austenite phase, taking into account two situations that magntic moment of excess Mn at the Sn sites (denoted as Mn_Sn_) is parallel or anti-parallel to that of Mn at Mn sites (denoted as Mn_Mn_). The parallel and antiparallel Mn_Sn_-Mn_Mn_ magnetic interactions are denoted as FM and AFM states, respectively. Figure 7(a) shows the curves of total energy *E* versus lattice constants for Ni_2_Mn_1.5_Sn_0.5_ austenite phase. It is seen that around equilibrium lattice constant, the AFM state of austenite phase is more stable than its FM state. Our results indicate that the magnetic moment of Mn_Sn_ in Ni_2_Mn_1.5_Sn_0.5_ austenite phase is antiferromagnetically coupled to the magnetic moment of Mn_Mn_. In the case of Ni_2_MnSn, it is well known that the magnetic interaction between Mn atoms is ferromagnetic. Thus, it is expected that with excess Mn substitution for Sn, the total magnetic moment of austenite phase decreases, which is agreement with the experiments. Moreover, the total energies of Ni_2_Mn_1.5_Sn_0.5_ with a variation of the tetragonal ratio (*E*-*c*/*a* curve) are calculated to reveal the phase transformation behaviors, as presented in Fig. 7(b). The *E*-*c*/*a* curves are calculated by keeping the volume constant at that of the *L*2_1_ cubic structure. In the whole range of *c*/*a*, the total energy of the structures under AFM state is lower than that under the FM state. For structures under the FM state, the energy minimum can be observed at *c*/*a* = 1 without any stable martensite phase being found. However, it is found that structure under AFM state, the energy minimum can be observed at *c*/*a* = 1.33, which corresponds to the *L*1_0_ structure of Ni_2_Mn_1.5_Sn_0.5._ This results show that the AFM austenite phase is unstable against the tetragonal distortion for Ni_2_Mn_1.5_Sn_0.5_ and undergoes the martensitic transformation to form AFM tetragonal martensite. Since both austenite and martensite are AFM state, saturation magnetization difference between two structural phases is small, which indicate weak MFIRMT in un-doped Ni_2_Mn_1.5_Sn_0.5_.

**Figure 8:**
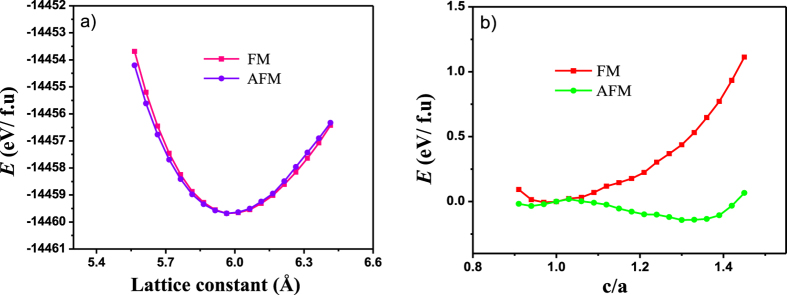
(**a**) Equation of states of Ni_1.75_Fe_0.25_Mn_1.5_Sn_0.5_ cubic phases for both parallel and antiparallel magnetic interactions. (**b**) The total energies *E* of Ni_1.75_Fe_0.25_Mn_1.5_Sn_0.5_ with a variation of the tetragonal ratio *c*/*a*.

**Figure 9:**
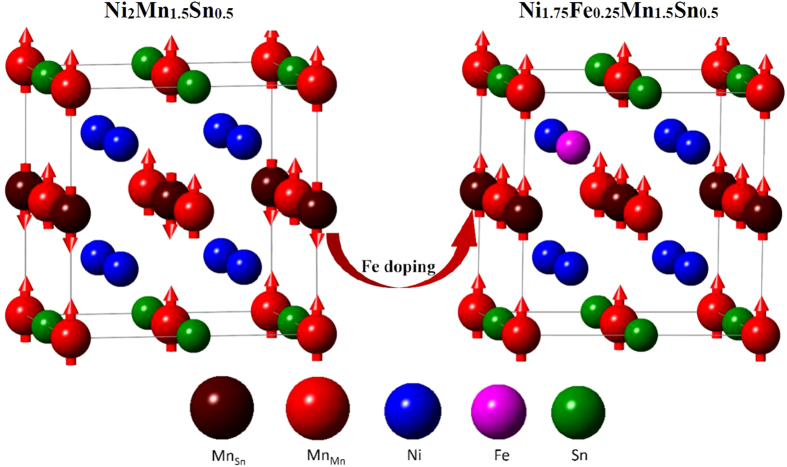
Illustration of substitution of Fe for Ni converting antiferromagnetic austenite to ferromagnetic state.

In the following, we focused on Fe doping Ni-Mn-Sn (Ni_1.75_Fe_0.25_Mn_1.5_Sn_0.5_). Figure 8(a) shows the equation of states of Ni_1.75_Fe_0.25_Mn_1.5_Sn_0.5_ austenite phase. It is worth noting that in contrast to the un-doped Ni_2_Mn_1.5_Sn_0.5_, the FM state of Ni_1.75_Fe_0.25_Mn_1.5_Sn_0.5_ is slightly more stable than the AFM state at equilibrium lattice constant, whereas the latter is more stable when the system is contracted. This result obviously indicates that substitution of Fe for Ni converts antiferromagnetic austenite to ferromagnetic state, as shown in Fig. 9 and hence increases the magnetization of austenite. Furthermore, the *E*-*c*/*a* curve for the Ni_1.75_Fe_0.25_Mn_1.5_Sn_0.5_ is plotted in Fig. 8(b). It can be found that Fe introduction do not change the energy behaviors along the variation of the *c*/*a*, but change the relative stability of the structure under the FM state to that under the AFM state. Thus, a competition between the FM and AFM states is clearly observed from the *E*-*c*/*a* curve. In the vicinity of *c*/*a* = 1, the structure under FM state is more stable than that AFM state. And the energy minimum can be reached at *c*/*a* = 1 with respect to the *L*2_1_ structure under FM state. With the *c*/*a* deviating from 1, the total energy of structure under FM state is increasing. However, for the structure under AFM state, the total energy decreased to the value lower than that of FM state, and reaching the energy minimum at the *c*/*a* > 1, which corresponds to the martensitic phase *L*1_0_ structure under the AFM state. From above analysis, we can reveal the origin of enhancing MFIRMT by Fe doping: the martensite *L*1_0_ structure for each system keeps the AFM state, whereas the antiferromagnetic austenite has been tuned to ferromagnetic state by substitution of Fe for Ni. Consequently, the magnetization of the austenite is significantly enhanced and the Δ*M* is effectively increased, which endows the Fe doped Ni-Mn-Sn system with enhancing MFIRMT, as observed in our experiment.

Moreover, according to the *E*-*c*/*a* curves, for both Ni_2_Mn_1.5_Sn_0.5_ and Ni_1.75_Fe_0.25_Mn_1.5_Sn_0.5_, the martensite phase can be reached as the structure with the energy minimum at the *c*/*a* > 1. It is known that the energy difference between the tetragonal martensitic and the cubic austenitic phases (Δ*E*) can be used to estimate the phase transformation temperature qualitatively, which usually increases with increasing Δ*E*^33^,^34^. Our calculated results show that Δ*E* for Ni_2_Mn_1.5_Sn_0.5_ and Ni_1.75_Fe_0.25_Mn_1.5_Sn_0.5_ are 22 meV/atom and 9 meV/atom, respectively. It can be seen that the system doped with Fe has smaller Δ*E* than that of the un-doped system, which indicates that substitution of Fe for Ni decreases the martensitic transformation temperature. This is in agreement with our experimental results. The calculations mentioned above may help in gaining an insight into the doping of Fe behavior in Ni-Mn-Sn and provide some theoretical aid to the material design.

## Conclusions

In the present work, we show the effect of substitution of Fe for Ni on microstructure, martensitic transformation, magnetic behavior and mechanical properties of Ni_50_Mn_39_Sn_11_ alloys. With the adding of Fe element, the crystal structure of the alloys has an evident change at room temperature. It transformed into the austenitic structure with high symmetry from martensitic structure with low symmetry. The martensitic transformation is clearly observed for the Ni_50−*x*_Fe_*x*_Mn_39_Sn_11_ (*x* = 0, 3, 4, 6 at.%) alloys, and the transformation temperatures decreased with increasing Fe addition in these alloys. The result fully shows that an appropriate amount of Fe atom substitution can really enhance the ferromagnetism of Ni_50_Mn_39_Sn_11_ alloy in austenite. It is also found that the proper amount of Fe addition significantly improves the compressive strength and the ductility of Ni_50_Mn_39_Sn_11_ alloy. The compressive strength increases from 215.0 MPa to 725.4 MPa and the compressive strain increases from 4.9% to 9.3% with increasing Fe content from 0 at.% to 4 at.%. The fracture type changes from intergranular fracture to transgranular fracture with increasing Fe content. Furthermore, we use first-principles calculations to gain insight into the origin of Fe doping on magnetic properties and martensitic transformation in Ni-Mn-Sn. Furthermore, using first-principles calculations, we found that enhancement of MFIRMT by Fe doping is originated from tuning antiferromagnetic austenite to ferromagnetic state by substitution of Fe for Ni. The martensitic transformation temperature can be quantified using the energy difference between the austenite and martensite phases. The Fe doping decreases the total energy difference between austenite and martensite of Ni-Mn-Sn, resulting in the decrease of its martensitic transformation temperature.

## Material and Methods

The nominal composition of the Ni_50−*x*_Fe_*x*_Mn_39_Sn_11_ alloys (*x* = 0, 3, 4, 6 at.%) were marked as Fe0, Fe3, Fe4 and Fe6, respectively. These alloys were prepared with high purity element nickel, manganese, tin and iron, with a purity level of 99.99%, 99.95%, 99.99% and 99.99%, by melting six times in a non-consumed vacuum arc furnace under argon atmosphere. The samples were annealed in vacuum quartz tubes at 1123 K for 12 h, and quenched in ice water for homogeneity.

The microstructure of the alloys was examined using a scanning electron microscopy (SEM) equipped with energy dispersive X-ray spectroscopy (EDS). The transformation temperatures were determined by differential scanning calorimetry (DSC) measurements with the TA-2920. The heating and cooling rates were 10 K/min. The compression tests were performed at room temperature on an Instron 5569 testing system at a crosshead displacement speed of 0.05 mm/min, and the size of the sample was 3 mm × 3 mm × 5 mm. Fracture cross-section was observed by SEM to study the dominant fracture behavior in this alloy system. The crystal structure at room temperature was determined by X-ray diffraction (Rigaku D/max-Rb with Cu K_α_ radiation). The magnetic properties were studied with a vibrating sample magnetometer (VSM).

Furthermore, we use first-principles calculations to reveal the origin of Fe doping on magnetic properties and martensitic transformation in Ni-Mn-Sn. The un-doped Ni_2_Mn_1.5_Sn_0.5_ and 6.25 at.% Fe doped Ni_1.75_Fe_0.25_Mn_1.5_Sn_0.5_ are employed in the calculations, which is close to the composition of samples in our experiment. All calculations have been performed based on the density functional theory, using CASTEP code^35^. The interaction between ions and electrons is described by ultra-soft pseudopotentials^36^. The spin-polarized generalized gradient approximation is used to describe the exchange correlation energy. The plane-wave cutoff energy is 400 eV and a (8, 8, 8) Monkhorst-Pack grid is employed to sample the Brillouin zone. For the cubic *L*2_1_ austenite of Ni_2_Mn_1.5_Sn_0.5_ and Ni_1.75_Fe_0.25_Mn_1.5_Sn_0.5_, 16 atoms unit cell is built with appropriate Sn atoms replaced by Mn atoms and Ni atoms replaced by Fe. The tetragonal martensite has been obtained by calculation of the stability of *L*2_1_ austenite with respect to volume-conserving tetragonal distortions. Then, the total energy and magnetic properties of both austenite and martensite have been calculated.

## Additional Information

**How to cite this article**: Tan, C. *et al*. Simultaneous enhancement of magnetic and mechanical properties in Ni-Mn-Sn alloy by Fe doping. *Sci. Rep.* **7**, 43387; doi: 10.1038/srep43387 (2017).

**Publisher's note:** Springer Nature remains neutral with regard to jurisdictional claims in published maps and institutional affiliations.

## Figures and Tables

**Table 1:** EDS results of annealed Ni_50−*x*_Fe_*x*_Mn_39_Sn_11_ (*x* = 0, 3, 4, 6 at.%) alloys.

	Matrix (at.%)				Second phase (at.%)			
	Ni	Mn	Sn	Fe	Ni	Mn	Sn	Fe
*x* = 0	49.8	39.1	11.1	0	—	—	—	—
*x* = 3	46.8	39.4	11.2	2.6	—	—	—	—
*x* = 4	46.3	39.2	11.7	2.8	44.1	38.9	6.3	10.7
*x* = 6	45.4	39.3	12.2	3.1	43.7	38.3	6.1	11.9

**Table 2:** The characteristic temperatures of Ni_50−*x*_Fe_*x*_Mn_39_Sn_11_ (*x* = 0, 3, 4, 6 at.%) alloys denoted as *M*_s_, *M*_f_, *A*_s_ and *A*_f_, respectively.

Composition	*M*_s_ (K)	*M*_f_ (K)	*A*_s_ (K)	*A*_f_ (K)
Ni_50_Mn_39_Sn_11_	386	365	394	400
Ni_47_Fe_3_Mn_39_Sn_11_	319	283	289	325
Ni_4*6*_Fe_4_Mn_39_Sn_11_	299	274	285	206
Ni_44_Fe_6_Mn_39_Sn_11_	222	206	214	232
